# Leukocyte-subset counts in idiopathic parkinsonism provide clues to a pathogenic pathway involving small intestinal bacterial overgrowth. A surveillance study

**DOI:** 10.1186/1757-4749-4-12

**Published:** 2012-10-19

**Authors:** R John Dobbs, André Charlett, Sylvia M Dobbs, Clive Weller, Mohammad A A Ibrahim, Owens Iguodala, Cori Smee, J Malcolm Plant, Andrew J Lawson, David Taylor, Ingvar Bjarnason

**Affiliations:** 1 Pharmaceutical Science, King's College London, Franklin-Wilkins Building, 150 Stamford Street, London SE1 9NH, UK; 2 The Maudsley Hospital, Denmark Hill, London, SE5 8AZ, UK; 3 Gastroenterology, King’s College Hospital, Bessemer Rd, London, SE5 9PJ, UK; 4 Statistics Unit, Health Protection Agency, 61 Colindale Avenue, London, NW9 5EQ, UK; 5 Clinical Immunology, King’s College Hospital, Bessemer Rd, London, SE5 9PJ, UK; 6 Laboratory of Gastrointestinal Pathogens, Health Protection Agency, London, NW9 5EQ, UK

**Keywords:** Pathogenesis of Parkinson’s disease, *Helicobacter*, Small intestinal bacterial overgrowth, Blood leukocytes, Natural-killer, T-helper, Neutrophils, Hypokinesia, Rigidity, Tremor

## Abstract

**Background:**

Following *Helicobacter pylori* eradication in idiopathic parkinsonism (IP), hypokinesia improved but flexor-rigidity increased. Small intestinal bacterial-overgrowth (SIBO) is a candidate driver of the rigidity: hydrogen-breath-test-positivity is common in IP and case histories suggest that *Helicobacter* keeps SIBO at bay.

**Methods:**

In a surveillance study, we explore relationships of IP-facets to peripheral immune/inflammatory-activation, in light of presence/absence of *Helicobacter* infection (urea-breath- and/or stool-antigen-test: positivity confirmed by gastric-biopsy) and hydrogen-breath-test status for SIBO (positivity: >20 ppm increment, 2 consecutive 15-min readings, within 2h of 25G lactulose). We question whether any relationships found between facets and blood leukocyte subset counts stand in patients free from anti-parkinsonian drugs, and are robust enough to defy fluctuations in performance consequent on short t½ therapy.

**Results:**

Of 51 IP-probands, 36 had current or past *Helicobacter* infection on entry, 25 having undergone successful eradication (median 3.4 years before). Thirty-four were hydrogen-breath-test-positive initially, 42 at sometime (343 tests) during surveillance (2.8 years). Hydrogen-breath-test-positivity was associated inversely with *Helicobacter*-positivity (OR 0.20 (95% CI 0.04, 0.99), p<0.05).

In 38 patients (untreated (17) or on stable long-t½ IP-medication), the higher the natural-killer count, the shorter stride, slower gait and greater flexor-rigidity (by mean 49 (14, 85) mm, 54 (3, 104) mm.s^-1^, 89 (2, 177) Nm.10^-3^, per 100 cells.μl^-1^ increment, p=0.007, 0.04 & 0.04 respectively, adjusted for patient characteristics). T-helper count was inversely associated with flexor-rigidity before (p=0.01) and after adjustment for natural-killer count (-36(-63, -10) Nm.10^-3^ per 100 cells.μl^-1^, *p*=0.007). Neutrophil count was inversely associated with tremor (visual analogue scale, p=0.01). Effect-sizes were independent of IP-medication, and not masked by including 13 patients receiving levodopa (except natural-killer count on flexor-rigidity). Cellular associations held after allowing for potentially confounding effect of hydrogen-breath-test or *Helicobacter* status. Moreover, additional reduction in stride and speed (68 (24, 112) mm & 103 (38, 168) mm.s^-1^, each p=0.002) was seen with *Helicobacter*-positivity. Hydrogen-breath-test-positivity, itself, was associated with higher natural-killer and T-helper counts, lower neutrophils (p=0.005, 0.02 & 0.008).

**Conclusion:**

We propose a rigidity-associated subordinate pathway, flagged by a higher natural-killer count, tempered by a higher T-helper, against which *Helicobacter* protects by keeping SIBO at bay.

## Background

Milestones in elucidating the aetiopathogenesis of Parkinson’s disease have been few and far between. We step back to consider the whole entity. Constipation featured in the original description [1]. Frequency of defecation deviates from that of controls three decades before median age of diagnosis [2], and infrequent bowel movements are associated with subsequent diagnosis [3]. Abnormal bowel function becomes more apparent post-diagnosis [2]. In contrast peptic ulceration has prodromal excess [4] and little-in-the-way of local aftermath [5]. Morphological and neurochemical changes associated with Parkinson’s disease are found in the enteric nervous system of oesophagus, stomach, duodenum and small- and large-intestine, in coeliac and para-verebral sympathetic ganglia and dorsal vagal nuclei [6-11]. Mitochondrial dysmorphology is seen in duodenal enterocytes in idiopathic parkinsonism [12]. This, if replicated in enteric neurones and/or myocytes, and associated with dysfunction [13], may provide a mechanism behind slow gastrointestinal transit.

What initiates and perpetuates slow transit? A viral primer cannot be excluded [14]: enteroviruses infect via the gastrointestinal tract and can have neurological sequelae; jejunal denervation is seen with human immunodeficiency virus infection, which can cause parkinsonism. In mice, intranasal inoculation with H5N1 influenza virus resulted in passage from enteric and dorsal root ganglia to brain stem. Ascent from here left a trail of microglial activation and dopaminergic degeneration [15]. Could *Helicobacter pylori*, the organism causally associated with peptic ulcer, affect transit? The *H. pylori* serum immunoblot antibody profile predicts abnormal bowel function in probands with idiopathic parkinsonism and their spouses [12]. In the presence of an anti-urease-B band, there was a four-fold increase in the odds of having abnormal function, irrespective of subject-group and urea-breath-test evidence of current infection. With an outer-membrane protein antibody band, there was a six-fold decrement.

Probands with idiopathic parkinsonism, aged ≤72.5 years, are twice [16], and siblings of probands three-times [17], as likely as controls to be seropositive for *H*. *pylori* anti-urease antibody. As in peptic ulcer/gastric carcinoma, there is no birth-cohort effect in antibody titre in probands in contrast to controls [16]. This is compatible with causality and/or progressive immunocompromise. In no disease where *H*. *pylori* is causal is it present in every case [18]. In Western populations, follow-up of cohorts tends to demonstrate a loss of *H*. *pylori*. This is generally attributed to widespread use of antimicrobials and acid-suppressants: low eradication rates cumulate with repeated exposure. The mainstay of anti-parkinsonian therapy, dopaminergic precursors, agonists and promoters, act locally and centrally to protect against experimental ulcers [19-21]. Whether therapeutic doses of dopaminergic agents suppress *Helicobacter* has not been explored, but agonists have been used to prevent duodenal ulcer relapse [22]. Danish population registers show increased prescription of *Helicobacter* eradication drugs in the 5 years prior to diagnosis of Parkinson’s disease [23], compatible with prodromal peptic ulcer [4]. Persistent infection might explain aggressive parkinsonism. Subjective motor assessment is, indeed, worse, relative to time since diagnosis, in Japanese probands with *H pylori*[24]. Parkinson’s disease has been linked to rural living and farm experience [25]. A mortality study of 26 US states found increased proportional mortality from Parkinson’s disease among livestock, but not arable, farmers [26]. Zoonotic-transmission of other gastric *Helicobacters* may contribute to the aetiopathogenesis.

*H*. *pylori* is an arbiter for progression of brady/hypokinesia in idiopathic parkinsonism [5,27]. Improvement in gait, in the year following successful blinded-active anti-*H*. *pylori* treatment [5], was replicated by open-active following initial placebo-randomisation. Gait plateaued over the subsequent two years. Improvement was independent of whether patients were untreated or receiving stable anti-parkinsonian therapy (levodopa use excluded to avoid iatrogenic fluctuations in performance). The effect is not related to infection-load: eradicating *H*. *pylori* detected only by molecular microbiology on culture-negative biopsies (‘low-density’ colonisation) occasioned similar improvement. Marked deterioration accompanied the natural experiment of eradication failure, even where persistence was at low-density. All failures were anti-nuclear antibody (ANA) seropositive. Moreover, ANA-positivity marked a poorer response to ‘successful’ eradication, perhaps indicating persistent undetected infection. Furthermore, irrespective of anti-urease ELISA seropositivity, the *H*. *pylori* serum immunoblot antibody profile against pathogenicity markers (cytotoxicity-associated gene-A product, vacuolating toxin-A and urease-B) is predictive of risk, severity and deterioration of idiopathic parkinsonism [28]. The underlying mechanism may be self-limiting auto-immunity. Autoimmunity is supported by the finding of HLA-DR risk loci [29,30]. It may have a peripheral (eg. skeletal muscle and cardiac mitochondria) as well as basal ganglia targets [12,14].

Improvement in brady/hypokinesia following *H*. *pylori* eradication was mirrored by an increase in objectively-measured flexor-rigidity: rigidity increased in year one post-eradication, plateaued over the subsequent two [5]. In a case study, this increase in rigidity coincided with onset of hydrogen-breath-test positivity for small intestinal bacterial overgrowth (SIBO), rigidity decreasing on regaining negativity [5]. Overgrowth may drive a subsidiary rigidity-associated pathogenic pathway, and be a relatively non-specific and dose-related player in perpetuating neuronal damage [14,27]. A 54% prevalence of glucose-hydrogen-breath-test positivity for SIBO is reported in Parkinson’s disease, versus 8% in controls [31]. Peripheral inflammation can evade or compromise the blood–brain barrier [32]. Overgrowth could provide a source of inflammation over a wide surface area with a strong haematogenous signal to microglia, as well as an afferent vagal [32]. (Gut-brain communication still occurs after vagotomy [33].)

Overgrowth is not an innocent bystander in the gastrointestinal tract: there is bloating and flatulence [31], and clouds of lysosomes are seen in duodenal enterocytes in relation to luminal bacteria (unpublished observation: A. Curry, SMD, RJD, IB). Long thin, often complex-branching, mitochondria are seen in duodenal enterocytes [14], rather than the protein arrays encapsulated by a double-membrane associated with *H*. *pylori*[12]. Overgrowth could drive homocysteine production and increase utilisation of vitamin B_12_ (a co-factor in homocysteine detoxification) [27]. Neither hyperhomocysteinaemia (43%) nor serum B_12_ concentration, in idiopathic parkinsonism, is explained by *Helicobacter* status. Since slow transit predisposes to reflux of colonic flora into the small-intestine, SIBO is likely to begin as a secondary phenomenon, but it may exacerbate gastrointestinal neuronal damage.

Here, we use surveillance data to explore the relationship of different facets of idiopathic parkinsonism to peripheral immuno-inflammatory activation, in the light of presence/absence of *Helicobacter* infection or of SIBO. Do any relationships between facets and blood leukocyte subset counts stand in patients free from anti-parkinsonian drugs, and are they robust enough to defy fluctuations in performance consequent on levodopa therapy? This builds on our demonstration of biological gradients of objective measures of facets on two systemic markers of inflammation, serum cortisol and tumour-necrosis-factor-α [34,35]. Reale et al. [36] have subsequently shown gradients of global scores of function and motor impairment on peripheral blood mononuclear cell production of cyto/chemokines and expression of nuclear factor κB. The setting is that, compared with controls, there is a relative lymphopenia in idiopathic parkinsonism, with an upward shift in the proportional distribution of natural-killer cells, a downward in B-cells [27]. T-helper and cytotoxic T-cell distributions are platykurtic (greater proportions than expected above and below reference ranges). Neutrophil counts tended to be higher. These findings were not attributable to anti-parkinsonian treatment. That of a relative lymphopenia confirmed previous smaller studies [37-40].

We also explore the relationship between *Helicobacter* and hydrogen-breath-test status in idiopathic parkinsonism. Gastric pathophysiology, conducive to peptic ulceration in the prodrome, might protect against colonisation of the small-intestine from above: gastrin secretion, evoked by antral-predominant gastritis, enhances the acid-barrier. Later, the barrier might be compromised by cytokine-mediated inhibition (local or central) of acid secretion [41] or by corporal gastric atrophy. After presentation, any *Helicobacter* gastritis is characteristically mild, atrophy absent [5], but inflammation associated with SIBO, secondary to caeco-ileal reflux, could progressively inhibit acid, with suppression of *Helicobacter* (as seen with proton pump inhibitors).

## Methods

### Surveillance data: patients and setting

Fifty-one consecutive patients with ‘clinically-definite’ idiopathic parkinsonism (IP) [42] and taking no or stable anti-parkinsonian medication were surveyed. The setting is a ‘gut-brain axis’ clinic, with emphasis on quantification of IP-facets to assess disease progression/response to routine interventions, and incorporating specialist neuropharmacology, gastrointestinal and immunological expertise. The sample is comprised of all patients eligible by the inclusion and exclusion criteria (Table 1). They were surveyed over a median follow-up of 2.8 (interquartile range 1.0, 3.6) years, limited by introduction of/change in anti-parkinsonian medication, in response to the doctor’s perception of the impact of disease on the patient’s life style and/or at the patient’s request.

**Table 1:** Inclusion and exclusion criteria for surveillance

**Inclusion**	
1.	Independently-living subjects with clinically-definite^a^ idiopathic parkinsonism
2.	Taking no or stable anti-parkinsonian medication
3.	Caucasian with English as first language and living in UK^b^
**Exclusion**	
1.	Secondary parkinsonism, ‘parkinsonism-plus’ syndromes and other wider clinical entities [70]
2.	Clinical depression [71], dementia [72] or other mental illness
3.	Other specific neurological condition
4.	Inflammatory bowel disease or history of major gastrointestinal surgery
5.	Other progressive or resolving disorders affecting physical ability or performance, or underlying incapacity sufficient to prevent assessments (e.g. use of walking aid)
6.	Cardiovascular/respiratory symptoms during normal activities
7.	UK MRC muscle strength score <4/5
8.	Arthropathy, mucsulo-skeletal disorder or overt abnormalities of, or history of orthopaedic surgery to, joints of spine or lower limbs
9.	Concurrent therapy with drugs which might be anti-dopaminergic or with hypnotics or sedatives
10.	Recent change in life situation (e.g. bereavement or change in marital status/domicile)

^a^Any combination of three of the cardinal features: resting tremor, rigidity, bradykinesia or impairment of postural reflexes. Alternatively sufficient two of the four features, with one of first three asymmetrical [42]. Responsiveness to a dopaminergic drug challenge not a requirement.

^b^To constrain ethnic and/or geographical influences.

Any assessments, blood sampling and breath-tests during acute inter-current illness were excluded from the analysis.

### Clinical measurements of outcome

The following disease facets are monitored routinely at each visit:-

### Brady/hypokinesia of gait

Distance-time gait indices are obtained using the shoestring device, a telemetric method suitable for routine assessment [43]. Rested patients walk, “at your own speed” over 18 m in a 1.85 m wide corridor. A second walk follows 5 min rest. Stride-length and speed are analysed: values refer to ‘steady-state’, editing out any build-up at start of walk, tail-off at end. Stride-length usefully defines treatment effects and, when corrected for relevant demographic/anthropometric characteristics, discriminates well between those with and without diagnosed parkinsonism [44].

### Upper limb rigidity

The supported forearm is moved horizontally, at a controlled velocity, through a 40° arc about the elbow [45]. The side judged more rigid at the initial assessment is monitored throughout. Swing duration is 1.3 s, the pause between varying (1 to 3 s) to reduce the effect of anticipation. One minute’s acclimatization precedes 2½ min recording. A computer interface unit measures the torque required for passive displacement against position (sampling interval 25 ms). Mean torque for each extending and flexing swing is calculated, grand means taken for resistance to extension (termed ‘flexor-rigidity’) and to flexion (‘extensor-rigidity’). Greater rigidity in flexor than in extensor muscles is characteristic of parkinsonism.

### Tremor of hands

A fixed camera films a plan-view of hands, resting semi-prone on the table in front of the seated patient [5]. It records any tremor over one minute before, and during, stress from repeating-back, in reverse order, series of spoken random-numbers. The archive of surveillance videos was audited by an independent assessor, blind to date and treatment, using a visual-analogue scale for each recording condition. (Scale was from 0, the most intrusive tremor witnessed between-subject, to 100, no tremor). Differences in this assessor’s ratings from those of two others are small (mean 3–6 %) and not significant [5].

### Postural abnormality

Instability at stance is measured by total angular displacement (sway) in the sagittal plane [46]: patients stand at ease, with eyes open for 1 min, then closed for 3 min. Mean coronal foot-separation at mid-swing, using the shoestring device, provides an objective measure of stability during walking [46,47].

### Psychomotor ability

Reaction time is measured as time taken to lift left or right index-finger from its touch-sensitive support [48]. Two seconds before the imperative, an alerting signal does, or does not, warn whether left or right is to be lifted. ‘Cognitive efficiency’ is measured by the ratio, unwarned⁄warned reaction time.

### Stance/gait videos

**Figure 1:**
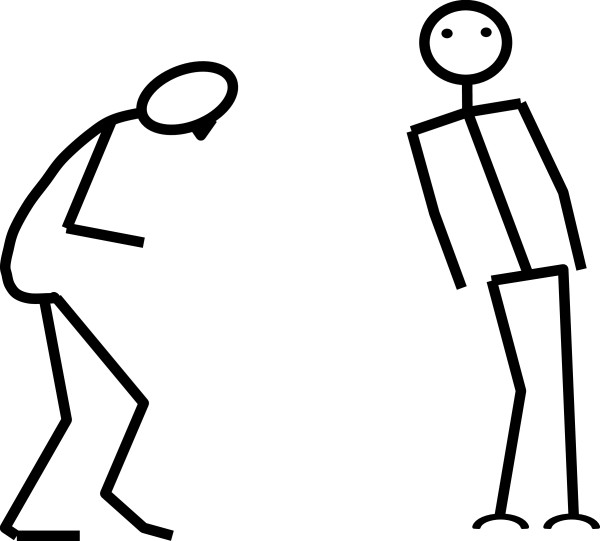
**Stickmen representing postural abnormalities in parkinsonism.** Simian (left), with flexion of neck, thoracic and lumbar spine, hips and knees, and hunched shoulders, is the most recognised. In coronal (right), there is lateral flexion of body and related abnormalities.

A ‘blinded’ independent auditor analysed anterior and lateral videos of standing (15 s) followed by walking (over 12.5 and 6.5 m, respectively). Lateral perspective is from the side on which rigidity is measured. Scales (Table 2 footnote) were constructed to describe brady/hypokinesia, simian and coronal postural abnormalities (Figure 1) and tremor. (Each scale was from 0, most severe abnormality witnessed in any patient, to 100, normality). The first three scales took equal account of items. Each item was composed of correlated groupings of formulated observations and/or their derivatives (e.g. inverse of cadence), which contributed equally. The scale for tremor during stance/walk simply took equal account of three observations. Observations were scored against set criteria (median 4 point scale, range 2–10 (derivative, inverse of cadence, excluded)). Chronbach’s alpha [49] showed satisfactory [50] within-scale consistency for the 12-item brady/hypokinesia (0.9), 13-item simian posture (0.7) and 3-observation tremor (0.9) scale. Consistency was not satisfactory for the 9-item coronal postural abnormality scale (0.4) overall, no better for its 3 items relating to ‘lower limb alignment’ or 6 relating to ‘head and body asymmetry’.

**Table 2:** Characteristics at start of surveillance period

**Characteristic**		**Mean (95% data interval) (n= 51 )**
Demographic	Age (years)	65 (48, 81)
	Gender (male/female)	32/19^a^
	Height (m)	1.69 (1.51, 1.86)
	Weight (kg)	76 (51, 102)
	Time since diagnosis (years)	6.5 (4.4, 8.9)^b^
	Anti-parkinsonian medication (no/yes)	17/34^a^
	Medication includes levodopa (no/yes) ^c^	21/13^a^
Blood cell counts	Total white cell count (10^9^/l)	6.4 (3.9, 1.3)
	Neutrophils (10^9^/l)	3.8 (1.9, 7.3)
	Lymphocytes (10^9^/l)	1.7 (0.9, 3.1)
	Mononuclear cell subset (/μl) T-helper (CD4+)	810 (345, 1903)
	T-cytotoxic (CD8+)	392 (118, 1305)
	B-cell (CD19+)	170 (44, 657)
	Natural-killer (CD16+CD56+)	258 (94, 705)
Brady/hypokinesia	Mean stride-length (mm)	1199 (795, 1602)
	Free-walking-speed (m/s)	1.16 (0.68, 1.64)
	Brady/hypokinesia scale (100 none, 0 worst)^d^	60 (55, 64)^b^
Rigidity	Mean torque to extend forearm (Nm x10^-3^)	589 (206, 1678)^e^
	Mean torque to flex (Nm x10^-3^)	286 (98, 830)^e^
	Ratio torque to extend/to flex	2.06 (0.80, 5.32)^e^
Tremor rating	Mean tremor whilst seated (100 none, 0 worst)	92.3 (62.8, 100)^b^
	at rest	100 (73.0, 100)
	under stress	90 (50, 100)
Postural abnormality	Tremor during stance/walk scale (100 none, 0 worst)^f^	98.3 (83.3, 100)^b^
	Mean body sway (°/min)	8.5 (3.7, 19.1)^e^
	with eyes open	6.1 (2.2, 17.4)^e^
	with eyes closed	9.2 (4.1, 2.4)^e^
	Mean foot separation (mm)	190 (141, 256)
	Simian posture scale^g^	79 (61, 96)^b^
	Coronal postural abnormality scale^h^	85 (72, 97)^b^
Psychomotor measures	Mean unwarned reaction time (ms)	518 (251, 1070)^e^
	Mean warned reaction time (ms)	322 (154, 672)^e^
Mean arterial pressure	Supine (mmHg)	95 (66, 123)
	Standing: mean over 3 min (mmHg)	97 (75,119)
Mean pulse rate	Supine (/min)	66 (48, 85)
	Standing: mean over 3 min (/min)	75 (54, 95)
Infection/overgrowth	Previous *Helicobacter* eradication (no/yes)^i^	26/25^a^
	*Helicobacter* status (+ve /-ve)	11^j^/40^a^
	Hydrogen breath-test status (+ve/-ve)	34^k^/17^a^

^a^Count.

^b^Median (interquartile range).

^c^as levodopa combined with extracerebral dopa-decarboxylase inhibitor ± catechol-O-methyl transferase inhibitor, none receiving combination as monotherapy. Other anti-parkinsonian medication used [selegiline, pramipexole, cabergoline, amantadine, trihexyphenidyl (low dose)] at steady state, and as evenly spaced as practicable with reference to t½, to avoid iatrogenic fluctuations in performance.

^d^12 items in scale: overall impression of brady/hypokinesia (4 observations), speed (3), stride including inverse of cadence (3), flow (2), ability to maintain ‘steady-state’ gait without progressive deterioration (2), symmetry of gait (2), pelvic swing (2), swing from hips (4), average of knee kick as leg swings past vertical (4), difference in kick between sides (2), average arm swing (4), and difference in swing between sides (2).

^e^Exponential of log_e_ transformed data.

^f^3 observations in scale, all relating to more rigid side: during walk from anterior perspective and from lateral, and at stance from lateral

^g^13 items: overall impression of simian tendency (4), festinant gait (2), head held flexed (3), chin projection (3), dropped jaw (1), hands behind back at stance (2), flexion at elbow (8), flexion at waist (4), compensatory shoulder retraction (1), flexion at hips (2), flexion at knees (4), inability to hyperextend at knee (4) and nature foot strike (4).

^h^9 items: 3 items for lower limb alignment (bow-legged (2), foot separation decrease (4), and foot inversion (6)), and 6 items for head and body asymmetry (body lean to worse side (3), body lean to best (3), shoulder elevation (3), difference in height of shoulders (2); head over to worse side (2) and head over to best (2)).

^i^Median (interquartile range) 1224 (1011, 2160) days prior to start of surveillance.

^j^2 had failed *Helicobacter*-eradication on urea-breath-test*,* and were retreated successfully.

^k^during surveillance, 42 were hydrogen-breath-test +ve at some point.

### Other measurements

Rested supine and standing (immediate and at 1 and 3 min) blood pressure and pulse are measured. Body weight is measured unshod, without outer clothing. Unshod height is obtained with buttocks and heels against a wall, but the patient otherwise relaxed.

### Blood cellular profile and autoantibody screen

Initially, a routine full blood count (XE2100, Sysmex UK Ltd., Wymbush UK), and mononuclear cell subset counts (FACSCalibur flow cytometer, Becton Dickinson, San Jose, California, USA, with four-colour fluorescent cell labelling using MultiTEST kit in TruCOUNT tubes) are obtained. The sum of the subset counts was validated by reference to ‘total lymphocytes’ in the full blood count. Serum is screened for ANA by indirect immunofluorescence using Hep2010 cells and rat stomach, kidney & liver composite tissue block (Euroimmun Biochip slides, Euroimmun UK, Pontypool, UK). Secondary antibodies are FITC-conjugated polyclonal rabbit anti-human IgG (Dako, Ely, UK). These standard immunological procedures are subject to the UK national external quality assurance scheme.

### Helicobacter status

Initially, *Helicobacter* status is checked by ^13^C]urea-breath-test (INFAI Ltd., York, UK) and/or stool antigen (Oxoid, Basingstoke, UK) in all patients. Infection is routinely confirmed by endoscopic-biopsy (with written informed consent) for histopathology and/or culture, and, if culture negative, detection of *H*. *pylori*-specific DNA [5]. Eradication therapy consists of one to two weeks’ proton pump inhibitor, plus two antimicrobials, selected according to *in vitro* sensitivities or with reference to clarithromycin susceptibility determined by molecular microbiology. Any history of intolerance is taken into account. Success is judged by the non-invasive tests at 6 weeks, confirmation by repeat endoscopic biopsy offered after at least 4 months.

### Hydrogen-breath-test status

Small intestinal bacterial overgrowth is screened for by the lactulose-hydrogen-breath-test in all patients, to monitor the need for/response to adequate fluid consumption and bulk/osmotic laxatives. A test consists of measuring breath-hydrogen concentration (Gastrolyser, Micro Medical Ltd., Rochester, UK) before and after (15-min intervals for 4 h) 25 G lactulose, following 24-h deprivation of dairy products (and medicinal lactulose) and a breakfast of 250 ml black tea/coffee or water. Status is defined by whether the meter manufacturer’s diagnostic cut–point (20 ppm increment) is exceeded by two consecutive readings within 2 h [51]. A prolonged test, with non-absorbable substrate, was chosen because of potentially impaired gastric-empting and intestinal transit [52]. Breath-hydrogen increases when lactulose reaches the colon, but false positives due to rapid oro-caecal transit [53] are very unlikely in IP. Use of glucose risks missing distal SIBO [54], since it is absorbed proximally.

### Statistical methods

Observational data are used to describe patterns of association between disease facets and blood leukocyte subsets. False-positives are not anathema in the structured approach to hypothesis generation described below, false negatives are: adjustment for multiple comparisons is inappropriate.

The association between a cell count and each physiological/cognitive ‘outcome’ measure was assessed in isolation, using a linear mixed effects regression model with outcome measure as dependent variable and cell count as independent. Account was taken of dependencies due to the physiological outcome being measured longitudinally: a random intercept was generated for each patient by inclusion of a patient random effect in the regression model. In contrast, cell counts and potential demographic covariates were taken to be invariable and measured once at the beginning of surveillance.

The regression analysis was first targeted at a ‘core group’ of 38 probands receiving no anti-parkinsonian medication, or medication other than levodopa. Independent variables, for which evidence of an association could not be discounted in the single variable analysis (*p*<0.2), were included in multivariable linear mixed models. A backwards elimination process was used to remove, in turn, the least significant independent variable, until all remaining exhibited a statistically important association. Discarded variables were then each included separately into the resultant multivariable model to ensure that their lack of association remained true. Cellular associations with outcome of *p*-value ≤0.05 were considered to be ‘statistically significant’. A between-subject analysis, using the average outcome measure in each patient, was used to substantiate associations of outcome measure and cell count, but sizes of effect given refer to the longitudinal multivariable models. (Estimated regression lines shown are from analyses which include all values for a longitudinal outcome, although, for presentation purposes, the points are restricted to an average per person.) Surrogacy of inter-related outcome measures in explaining cellular associations was examined. Longitudinally-assessed *Helicobacter* and hydrogen-breath-test status was included in the final models to determine whether they might explain any associations between outcome and cell count. Multivariable models were fitted in the 17 ‘untreated’ patients alone and in the ‘entire’ group of 51 (including those receiving the short t½ medication, levodopa) to assess the consistency of associations in these contexts.

Similarly, single variable analysis and multivariable generalised linear mixed modelling were used to assess associations between hydrogen-breath-test status and cell counts.

A natural logarithmic transformation to approximate ‘Normality’ was performed on variables with a positively-skewed distribution. Assumptions of Normally distributed random effects and residuals were checked graphically using normal quantile plots. Combined scaled measurements obtained from videos had approximately Normal distributions.

## Results

### Characteristics of patients with idiopathic parkinsonism

Table 2 gives baseline demographic and haematological data, outcome measures, and status with respect to *Helicobacter* and hydrogen-breath-test for the entire group.

Hydrogen-breath-test positivity was associated inversely with *Helicobacter* positivity (OR 0.20 (95% CI 0.04, 0.99), p=0.05). Hydrogen-breath-test positivity was unrelated to receipt of anti-parkinsonian medication other than levodopa, but directly associated with levodopa receipt (3.50 (1.01, 12.14), p=0.05).

### Cellular association with breath-hydrogen

**Figure 2:**
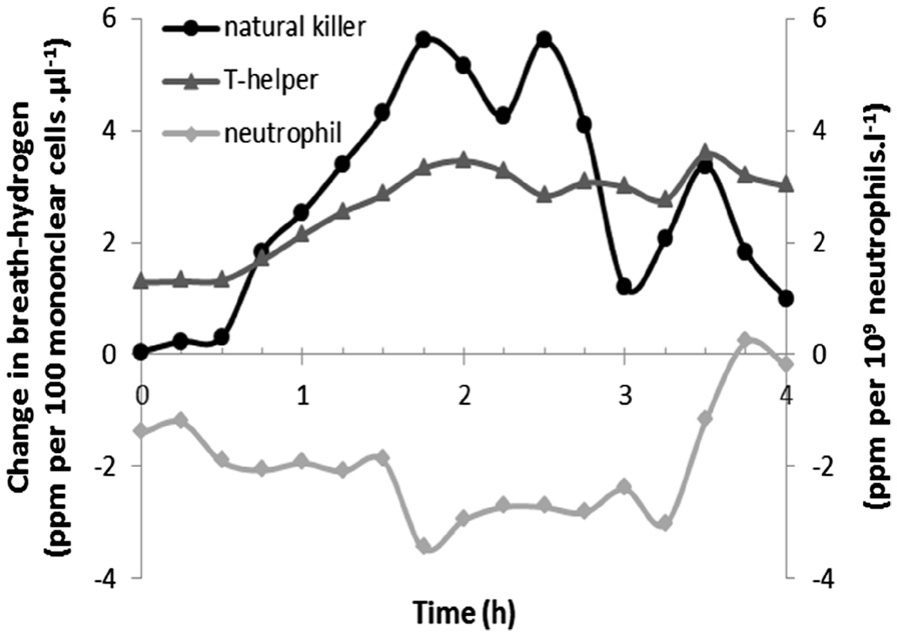
**Relationship between breath-hydrogen concentration and blood leukocyte subsets over time after lactulose administration.** Average increase in breath-hydrogen over baseline (343 tests; 15 min sampling intervals over 4h) is standardised for leukocyte count. Test positivity in the first 2h was associated with higher natural killer and T-helper mononuclear counts, lower neutrophils. Subsequently, breath-hydrogen was associated with T-helper count, tended to be with natural-killer (3 (95% CI 0, 4) & 5 (0, 11) ppm per 100 cell.μl^-1^ increment, respectively, p=0.02 & 0.06), but was not associated with neutrophil.

Of the entire group, 67% were classified as positive on the basis of the first 2 h of the initial hydrogen-breath-test, 82% being positive at some point during surveillance. Positivity was not associated with total lymphocyte, cytotoxic T-cell or B-cell counts, but with higher natural-killer (CD16+56+) (*p*=0.01) and T-helper (CD4+) (*p*=0.05) counts, and a lower neutrophil polymorphonuclear leukocyte count (*p*=0.02). Our multivariable explanation contained these (odds ratios for positivity: 1.7 (95% CI 1.2, 2.5) & 1.2 (1.0, 1.3) per 100 mononuclear cells.μl^-1^ increment, respectively; 0.65 (0.48, 0.89) per 10^9^ neutrophils.l^-1^, *p*=0.005, 0.02 & 0.008) and age (*p*=0.04). Figure 2 shows that the association of breath-hydrogen with T-helper count is sustained to 4 h. That with natural-killer count tended to be, but the neutrophil association was lost.

### Cellular associations with clinical measurements

We present associations, meeting the set criteria, of leukocyte subsets implicated above (first natural-killer, second T-helper, third neutrophil), followed by any associations of other subsets studied (total lymphocyte, cytotoxic T-cell or B-cell), with:-

**Figure 3:**
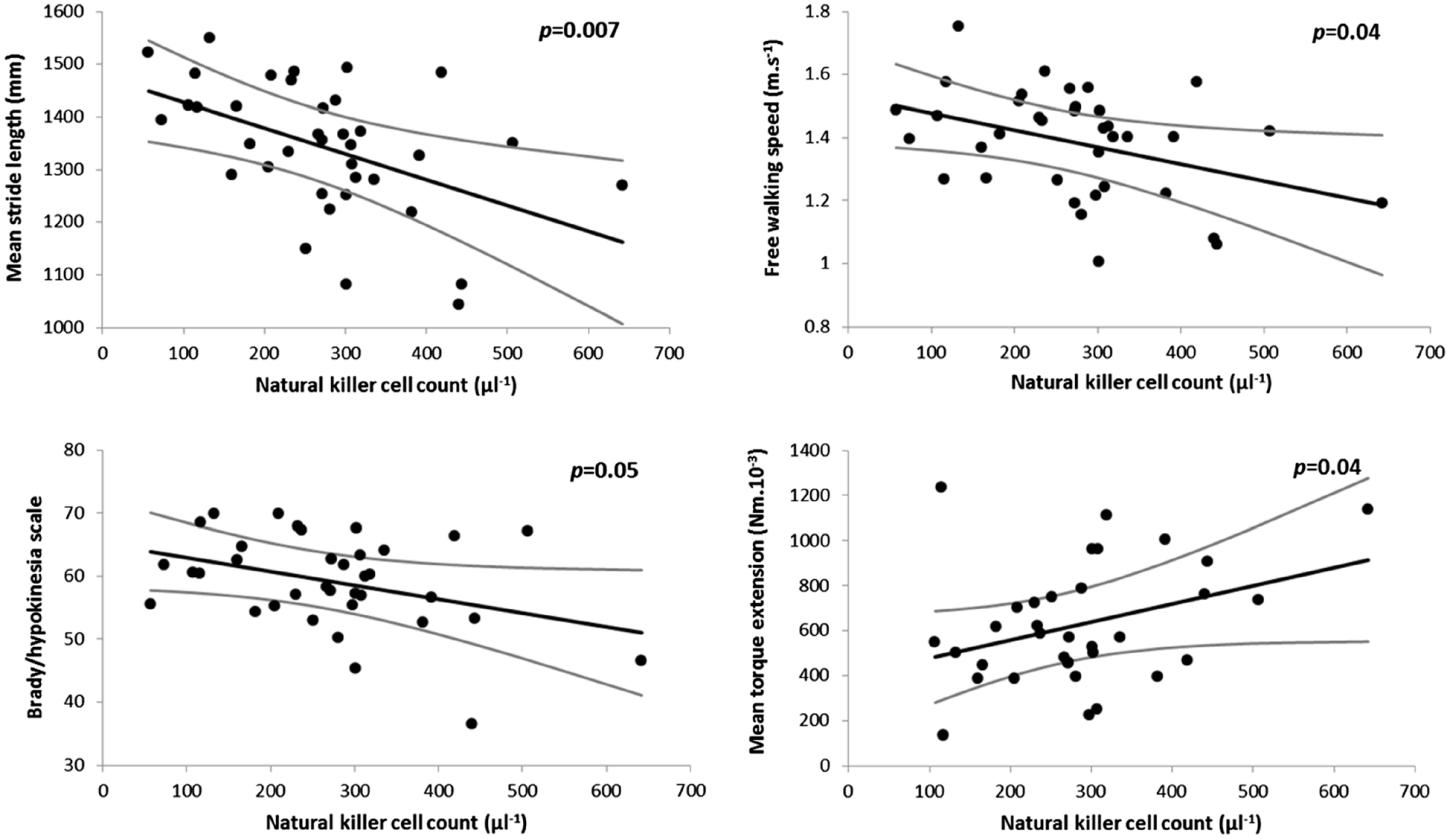
**Relationship of brady/hypokinesia and flexor-rigidity to natural-killer count in core group, after adjustment for demographic characteristics and, in the case of flexor-rigidity, for T-helper count.** (Standardised, as appropriate, to no background medication, age 65 years, height 1.7 m, time since diagnosis 6 years, T-helper count 1000 μl^-1^.) Regression line and 95% CI are shown for stride-length, free-walking-speed, the brady/hypokinesia scale and flexor-rigidity on natural-killer count. Although all values for a longitudinal outcome were used to estimate regression lines, the points shown are the average per person. (Brady/hypokinesia scale: 0 = marked impairment on video, 100 = none).

*Brady/hypokinesia and rigidity.* In the core group of 38 patients (untreated plus those receiving anti-parkinsonian medication other than levodopa), single variable analysis revealed significant relationships of natural-killer count to mean stride-length, free-walking-speed and flexor-rigidity (*p*=0.007, 0.02 & 0.02): the higher the count, the worse the facet. Size of effect remained clinically important after correction for relevant demographic covariates (Additional file 1: Table S3, Part a, Figure 3). Count was not related to extensor-rigidity or to the difference, flexor minus extensor. The association of natural-killer count with brady/hypokinesia was even captured from video by the brady/hyokinesia scale (Figure 3), just as were the additional effects of medication status and time since diagnosis (*p*=0.01 in each case).

**Figure 4:**
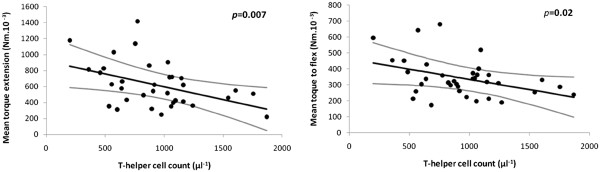
**Relationship of rigidity to T-helper count in core group, after adjustment for demographic characteristics, and, in the case of flexor-rigidity, for natural-killer count.** (Standardised, as appropriate, to time since diagnosis 6 years, body weight 80 kg, natural-killer count 250 μl^-1^.) Regression line and 95% CI are shown for flexor- and extensor-rigidity on T-helper count.

T-helper count ‘modulated’ the effect of natural-killer count on rigidity (the higher the count, the less rigidity), but did not contribute to the explanation of brady/hypokinesia by natural-killer count. It was associated with flexor-rigidity both before (*p*=0.01) and after adjusting for natural-killer count and covariates, with extensor only after covariate adjustment (Figure 4).

**Figure 5:**
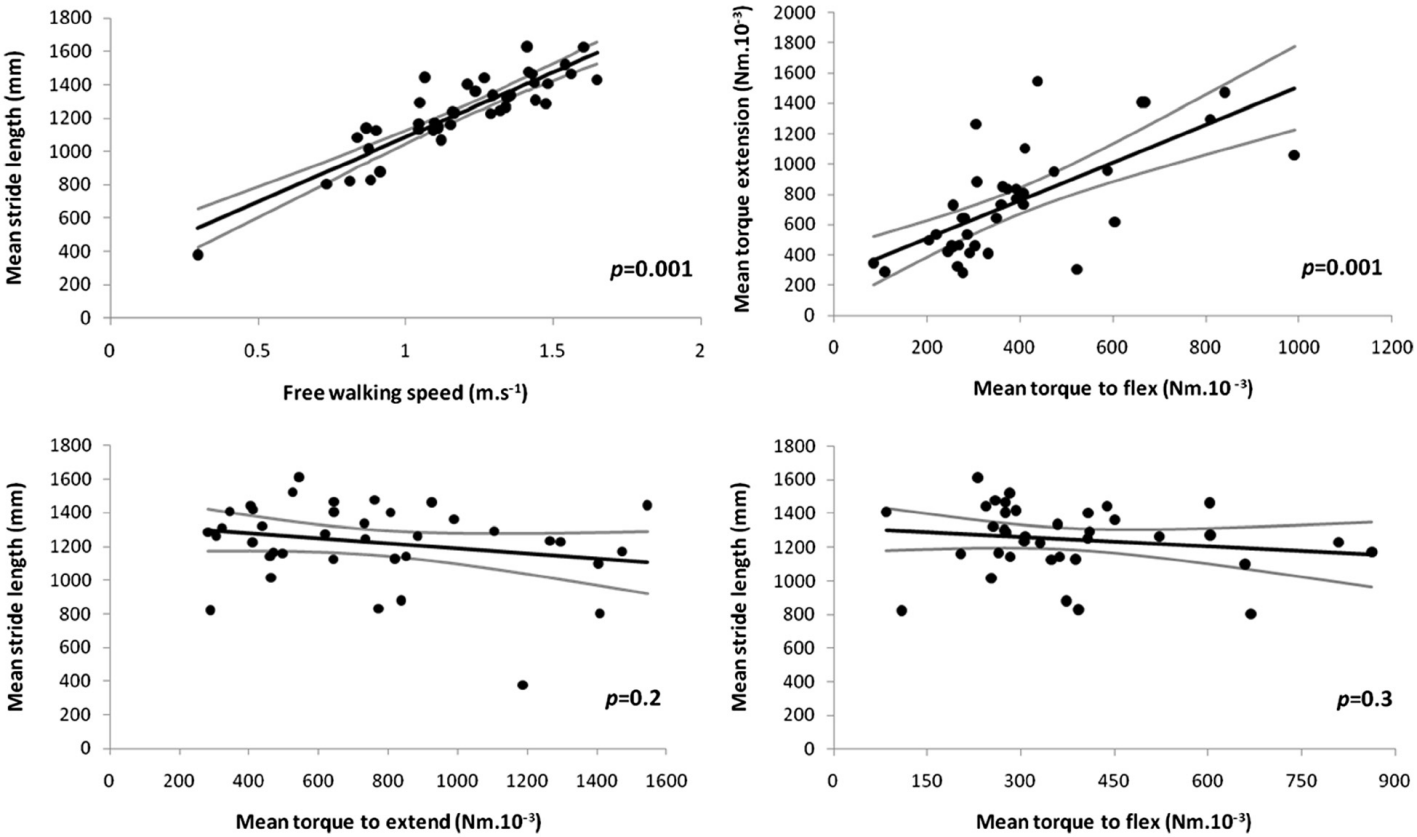
**Relationship, in core group, of hypokinesia (stride-length) to bradykinesia (speed) and flexor-rigidity to extensor, and independence of hypokinesia from rigidity.** No adjustment is made for demographic covariates. Regression line and 95% CI are shown for stride on speed (upper left graph - point with co-ordinates of stride 378 mm & speed 0.3 m.s^-1^ not influential); flexor-rigidity on extensor (upper right); stride on flexor-rigidity (lower left); stride on extensor-rigidity (lower right - after removal of an influential point: co-ordinates stride 378 mm & rigidity 990 Nm.10^-3^).

Mean stride-length and free-walking-speed were highly associated, flexor- and extensor-rigidity less tightly so, whilst stride showed no association with flexor- or extensor-rigidity under the test conditions (Figure 5). Accordingly, the association of stride with natural-killer count was not confounded by including flexor-rigidity in the multivariable model, nor were those of flexor-rigidity with natural-killer and T-helper counts confounded by including stride-length. Cellular associations held after allowing for the potentially confounding effect of hydrogen-breath-test or *Helicobacter* status. *Helicobacter* positivity was, however, associated with an additional shortening of stride and reduction in speed (by 68 (24, 112) mm & 103 (38, 168) mm.s^-1^ respectively, *p*=0.002 in each case), but it had no effect on rigidity.

Associations with cell counts remained after adjustment for presence/absence of anti-parkinsonian medication in the core group (Additional file 1: Table S3, Part a). Indeed, size and significance of effects were retained in the untreated group (Additional file 1: Table S3, Part b). In the entire group, which included patients receiving levodopa, size of effect of natural-killer count on objective measures of brady/hypokinesia was similar to that in the core (Additional file 1: Table S3, Part c), definition of effect on stride sharper in the core. Both size of effect and significance of the association between natural-killer count and flexor-rigidity were lost in the entire group. Those of T-helper count with both flexor- and extensor-rigidity were as strong as in the core. It should be noted that levodopa was not used as first-line treatment: stride tended to be shorter (by 128 (268, -12) mm, *p*=0.07) in those receiving it, rigidity was similar.

### Mean arterial pressure

**Figure 6:**
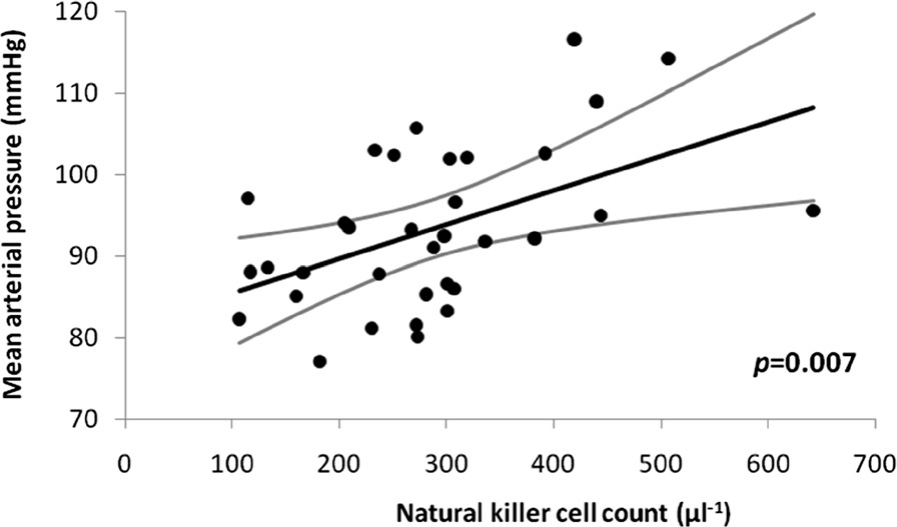
**Relationship of supine mean arterial pressure to natural-killer count in core group, after adjustment for demographic covariates (standardised to age 65 years, time since diagnosis 6 years, body weight 80 kg).** Regression line and 95% CI are shown. On inclusion of flexor-rigidity in multivariable model, cellular association remained 3.4 (0.4, 6.5) mmHg per 100 cells.μl^-1^ increment, p=0.03), and an additional small effect on pressure was seen (increase of 3.4 (0.8, 6.0) mmHg per 500 Nm.10^-3^ greater rigidity, p=0.01).

Hypotension is a feature of idiopathic parkinsonism. However, in the core group, single variable analysis revealed a significant relationship of natural-killer count to supine mean arterial pressure (*p*=0.02): the higher the count, the higher the pressure. This effect remained after correction for relevant demographic covatiates (Additional file 2: Table S4, Part a, Figure 6). Its magnitude was equivalent to the predicted increase in pressure with 10 years of age or 10 kg of body weight, or decrease over 4 years from diagnosis. The association of natural-killer count with pressure was not a surrogate for that with brady/hypokinesia or flexor-rigidity. Any association between cellular profile and standing pressure was masked by variability in orthostatic response (mean difference from supine −4 (data interval −18, 10) mmHg).

The natural-killer count association with pressure was not confounded by hydrogen-breath-test or *Helicobacter* status. Neither status had an additional effect on pressure. Size of effect of natural-killer count on pressure was independent of anti-parkinsonian medication in the core group, similar in the untreated and entire (Additional file 2: Table S4, Parts a, b and c). However, in the untreated, it did not reach statistical significance.

### Tremor

**Figure 7:**
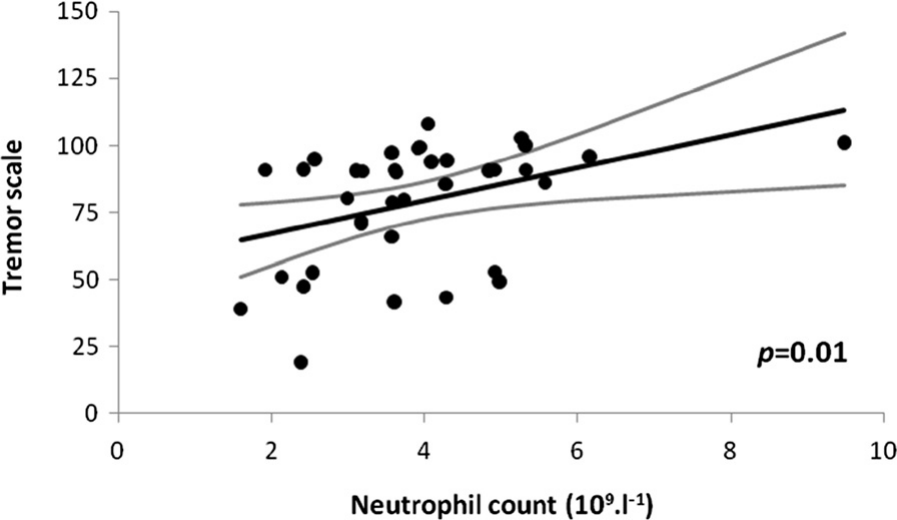
**Relationship of mean tremor whilst seated to neutrophil count in core group, after adjustment for time since diagnosis (to 6 years).** Regression line and 95% CI are shown. Outlier for neutrophil count (9.48 x10^9^.l^-1^) not influential (size of effect after exclusion: decrease in tremor of 7.9 (95% CI 1.3, 14.5) units per 10^9^.l^-1^ cell increment, p=0.02). Tremor scale: 0 = most intrusive, 100 = no tremor (N.B. 3 values >100 (i.e. 101, 103, 108) consequent on adjustment for time since diagnosis).

In the core group, single variable analysis revealed a significant relationship of neutrophil count to the mean score for tremor whilst seated (*p*=0.03): the lower the count, the greater the tremor. This small but significant effect remained after correction for the relevant demographic covariate, time since diagnosis (Additional file 3: Table S5, Part a, Figure 7), and was independent of condition, rest or stress. Its magnitude was equivalent to the predicted increase in tremor with 5 years’ time lapse. One might expect a stiff arm to tremble less. Indeed, although rigidity held no surrogacy for tremor in the neutrophil association, flexor-rigidity tended to have an additional effect on tremor: the greater the rigidity, the less the tremor (by 3 (95% CI 0, 6) units per 500 Nm x10^-3^, *p*=0.09).

The neutrophil association with tremor was not confounded by hydrogen-breath-test or *Helicobacter* status. However, *Helicobacter* positivity had an additional effect: tremor was less with positivity by 6.3 (0.5, 12.2) units (*p*=0.03). Size of effect of neutrophil count on tremor was independent of anti-parkinsonian medication in the core group, retained in the untreated and not masked in entire (Additional file 3: Table S5, Parts a, b and c). However, in the untreated, it reached significance only at the 0.1 level.

No cellular association was seen with postural tremor while standing and walking, the neutrophil association with seated tremor remaining after including postural tremor in the model.

### Postural abnormality

**Figure 8:**
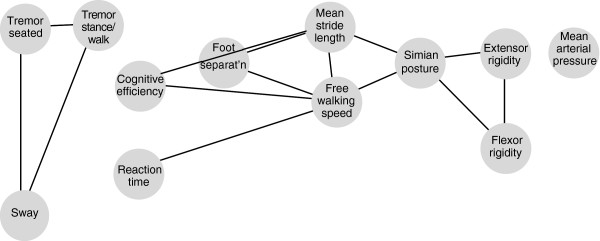
**Between-patient relationships of clinical surveillance tools in core group.** Outcome measures are represented by discs. Single variable associations, of strength p≤.05, are represented by lines ending at corresponding disc perimeters. The closer the discs, the more variance explained: line length is inversely proportional to variance in one outcome explained by the other. Relationships resting on influential points, or not holding in mixed-effects models, were dismissed. Absence of connecting lines means lack of association. Greatest variance represented by a connecting line between a pair of outcomes (78%) is for free-walking-speed and mean stride-length, least (13%) for reaction time and speed. Variance explained by multiple associates can, of course, be cumulative (e.g. variance in simian posture rating explained individually by flexor- and extensor-rigidity, stride and speed is 30, 53% 40% & 40%, respectively, but together they explain 68%). Principle component analysis of 13 items making up simian posture scale indicated a first component, average score for 9 items, with a greater within-scale consistency than the original (Chronbach’s alpha 0.8 cf 0.7), and retaining association with brady/hypokinesia and rigidity. Other components could not be interpreted simply in terms of remaining items: 3 compensatory strategies (chin projection, hands behind back at stance, shoulder retraction), and dropped jaw. These were not associated with brady/hypokinesia and rigidity, and showed no association *inter se*.

There is a case for a B-cell (CD19+) association with posture ratings, but it falls short of the set criteria. Simian posture score showed a significant association with B-cell count in the core group, only after adjustment for time since diagnosis: the higher the count, the better posture (by 2.1(0.1, 4.1) units per 100 cells.μl^-1^ increment, *p*=0.03). Size of effect is set in context by a 25 to 75^th^ centile range from just 76 to 87. The strength of the cellular association with simian posture (*p*=0.01, after adjustment for hypokinesia and rigidity (see Figure 8 legend)) was not confounded by incorporating coronal posture into the model (*p*=0.004). Size and significance of effect were retained in entire group, lost in the relatively small untreated group.

Single variable analysis did reveal a relationship of B-cell count to coronal posture rating (*p*=0.05): the higher the count, the better posture. Significance was similar (*p*=0.06) after correction for relevant covariates, time since diagnosis and weight. Rigidity held no surrogacy for coronal posture in the B-cell association, but its inclusion did have a small additional effect (*p*=0.001): the greater extensor-rigidity, the worse posture. Size of effect was retained in untreated (p=0.1), and entire (p=0.07) groups.

Neither hydrogen-breath-test nor *Helicobacter* status held surrogacy for the B-cell associations or had any direct effect on posture ratings. No cellular association was identified for objective assessments of standing body sway and ambulatory foot separation on single variable analysis.

### Lack of association with psychomotor ability

There was no single variable association of reaction time (mean of unwarned and warned) or cognitive efficiency with any of the leukocyte subsets.

### Overview of surveillance tools

Figure 8 gives an empirical overview of inter-relationships of the outcome measures between patients, using single-variable analysis.

Three cardinal signs, brady/hypokinesia, rigidity and tremor, are independent of each other under test conditions. The direct relationship between mean stride-length and free-walking-speed is close, as is that between tremor measured whilst seated and during stance/walk. That between flexor- and extensor-rigidity is not as close.

Tremor is weakly related to body sway: the worse the tremor, the greater the sway. Ambulatory coronal foot separation has a moderate association with brady- and hypo-kinesia: the narrower the separation, the worse gait. Simian posture rating is moderately related to brady- and hypo-kinesia, and to rigidity: bad posture goes with poorer gait and worse rigidity. Cognitive efficiency shows a weak direct association to stride and speed, reaction time a weak inverse association with speed: good psychomotor performance under test conditions is reflected in gait.

Supine mean arterial pressure stands independent of the other outcomes.

### Helicobacter infection and small intestinal bacterial overgrowth in spouses

Spouses of 15 patients had requested screening for *Helicobacter* and SIBO: two-thirds (67 (exact binomial 95% CI 38, 88) %) were hydrogen-breath-test positive. One of these and two of the negatives were urea-breath-test positive.

## Discussion

Here we focus on the aftermath of *H. pylori* infection. In the gut/brain axis clinic, nearly three-quarters of the IP-patients surveyed had evidence of current or past infection on entry. Half had undergone successful eradication, a median of 3 years before. Two-thirds were lactulose-hydrogen-breath-test positive initially, four-fifths at some time during surveillance.

Our approach to aetiology/pathogenesis is to start from clinical clues. Statistical modelling of observational data is used to generate hypotheses. Having found subjective global clinical scores to be blunt instruments [55], we have embraced valid, sensitive, specific and reliable measures of disease facets. To avoid incorrect inferences, we endeavour to understand what is being measured, its variability and what influences it (i.e. confounding influences and bias), and explore effect modification (by interaction or directly). Use of objective measures brings economy of sample size. The pattern of cellular associates reported emphasises that facets may have different, non-coincident driving forces.

### Biological gradients

Bradford Hill [56] wrote “The clear dose–response curve admits of a simple explanation”, causality. “Often the difficulty is to secure some satisfactory quantitative measure of the environment which will permit us explore this dose–response.” We identify biological gradients of facets of IP on blood leukocyte subset counts, and find the same group of subsets linked to SIBO. The simplest biologically plausible explanation is that the counts represent driving/ameliorating forces on the facets, and that SIBO is their source. The ‘Bradford Hill predicament’ is to secure which components and/or associates of SIBO might affect the inflammatory response. However, a complex causal pathway does not preclude interim clinical solutions. In IP, hydrogen-breath-test positivity indicates the need to assess faecal overload, particularly in caecum and ascending colon, and, where appropriate, to institute adequate fluid consumption and bulk/osmotic laxative therapy. Re-accumulation of overload is likely: enterokinetic agents have a potential role, particularly if the colon is dyskinetic with an element of retrograde propulsion [57].

Outcome associations with three leukocyte subsets withstood rigorous statistical criteria. Under test conditions, three facets were associated with natural-killer cell (CD16+56+) count: brady/hypokinesia, flexor-rigidity and higher mean arterial pressure. A higher count may be accompanied by higher serum cortisol [34] with fluid retention, whereas (orthostatic) hypotension would be expected from ‘cold’ degeneration of sympathetic ganglia and brain stem noradrenergic nuclei. T-helper (CD4+) count was associated inversely with flexor-rigidity, neutrophil count inversely with tremor, as if mounting these immune responses were protective. Associations were independent of anti-parkinsonian medication.

In IP, SIBO has systemic consequence. Moreover, the above three out of the six leukocyte categories studied (total lymphocytes and neutrophils from full blood count; CD4+, CD8+, CD19+ and CD16+56+ mononuclear subsets) were associated with hydrogen-breath-test positivity, suggesting a mechanistic link. Hydrogen-breath-test status did not *per se* account for leukocyte associations with disease facets. Other measures of SIBO, a component or non-bacterial associate of SIBO, or imbalance in intestinal microbiota might. Hydrogen-breath-test positivity, like severity of IP manifestations, was related to higher CD16+56+ count and lower neutrophils, but, unlike severity, to a higher CD4+ count. The CD4+ subset includes regulatory T-cells which can inhibit effector mechanisms of natural-killer cells. A single rigidity-provoking taxon might evoke an increase in CD16+56+ count, a decrease in CD4+. For example, escape [58] of a viral cause of slow transit may cause an increase in CD16+56+ count and, were it lymphotropic, suppression of CD4+. Alternatively, different components/associates of SIBO, with different cellular responses, might have opposite effects on rigidity.

Breath-hydrogen concentration from 2 to 4 h after lactulose reflected the earlier T-helper and natural-killer count associations, but not the neutrophil. Chronic bacterial overload may suppress neutrophils, overload in the more distal small-intestine not having the same capacity to suppress neutrophils as that in the proximal. A degree of immune tolerance to refluxed colonic microbiota might be acquired.

### Definition of phenotype

The independence of the measures of brady/hypokinesia, rigidity, tremor and mean arterial pressure described provides a foundation on which to build a wider description of IP. Further measures should be complementary, not congruous.

It is remarkable that the relationship between natural-killer cells and brady/hypokinesia was robust enough to be captured by video assessment. However, the brady/hypokinesia scale becomes redundant in the face of rapid objective quantification. Focusing, in a formal protocol, on tremor whilst seated captured the neutrophil association, observing incidental tremor during walking did not. Although both simian and coronal posture ratings tended to capture a B-cell association, they were unrelated in single variable analysis. The association with simian posture persisted after large scale adjustment for rigidity (on the worse side) and brady/hypokinesia. Bilateral quantification of rigidity is a next step towards better definition of these B-cell associations. Coronal postural abnormality is a manifestation of disease asymmetry and compensation for this.

### Assimilation of findings into potential pathogenic pathways

To consider causality in terms of single question steps, arising in unique linear order, denies the multi-step, multi-factorial nature of chronic disease. Not until a large number of observational and interventional findings have been assimilated will their position within the causal pathway become more certain. It cannot be assumed that disease facets will progress in parallel. The goal is stratification of pathogenic pathways into those central to disease initiation and the subsidiary, which may/may not be activated.

Our work (Table 3) points to three pathogenic pathways:- (i) A brady/hypokinetic response to *Helicobacter* infection [5]. (ii) A rigidity-associated pathway, linked directly to natural-killer count, inversely to T-helper. (iii) A tremor-associated pathway linked inversely to neutrophil count. Brady/hypokinesia appears to have two pathogenic components: its association with *Helicobacter* positivity was additional to that with natural-killer count. Evidence for a fourth pathway does fall short of our rigorous statistical criteria, but better posture with a higher B-cell count suggests disease attenuation in the immunocompetent.

**Table 3:** **Summary of evidence that *H. pylori *infection and small intestinal bacterial overgrowth drive facets of parkinsonism**

**Gastrointestinal disorder**	**Evidence**	**Outcome**
*H. pylori* infection	Double-blind, placebo-controlled, randomised efficacy study of eradication.	Differential effects with improvement in hypokinesia and worsening rigidity over year post-eradication and subsequent (2-year) plateau. Overall: net improvement [5].
	Surveillance	(i) Detrimental effect of *Helicobacter* positivity on hypo- and bradykinesia (adjusted for natural-killer count) [**Results**: *Cellular associations with clinical measurements*].
		(ii) Improvement in hypokinesia followed *Helicobacter-*eradication, but not antimicrobials given for other indications (*i.e.* indication specific) [73].
Small intestinal bacterial overgrowth	Surveillance	(i) Same three leukocyte subset associated with hydrogen-breath-test positivity and facets (biological gradient of rigidity on natural killer and T-helper counts; brady/hypokinesia on natural killer; tremor on neutrophil) [**Results**: *Cellular association with breath-hydrogen* &*Cellular associations with clinical measurements*].
		(ii) Increased rigidity followed antimicrobials for indications other than *Helicobacter* (*i.e.* not indication-specific), suggesting alteration in intestinal microbiota as a player [73].

The seemingly self-perpetuating microglial activation of IP [59] may actually be a response to a continuous stimulus, commonly SIBO. This does not exclude exacerbation by other inflammatory stimuli. Patients frequently report transient worsening of parkinsonism with intercurrent illness/its treatment. Moreover, the adaptive immune response in substantia nigra in Parkinson’s disease [60], and the presence of peripheral immune cells (as well as Lewy bodies) in therapeutically-useful dopamine cell brain implants [61], fit with a peripheral immune process driving neuronal damage.

High prevalence of SIBO in IP has been shown by hydrogen-breath-tests using different substrates, lactulose and glucose. The slightly lower prevalence of positivity using the absorbable sugar [31] may represent missed distal colonisation. We demonstrate an inverse association between hydrogen-breath-test and *Helicobacter* status. Since *H. pylori* is usually acquired in infancy, it is reasonable to postulate that any protection is by *Helicobacter* against SIBO. Whether the acid barrier and/or immune regulation determine such protection needs elucidation. The stability in anti-parkinsonian medication status over the surveillance period (median 2.8 years) may be a consequence of *H. pylori* eradication and attenuating SIBO by maintaining gastrointestinal transit.

Our usage of levodopa (as an adjuvant where functional impairment no longer responds adequately to other anti-parkinsonian medication) was associated with hydrogen-breath-test positivity. Meeting our indication for levodopa might coincide with worsening constipation [2], with more caeco-ileal reflux. Indeed, in a group of patients exposed only to levodopa combinations [31], hydrogen-breath-test positivity was linked to functional staging and severity, with no additional effect of levodopa dosage (average 800 mg/day).

### Dopaminergic status as a determinant of immune response

It might be argued that the cellular associations with facets of parkinsonism simply reflect the influence of dopaminergic status on leukocytes. Dopamine has been implicated in immunoreguation [62,63]. Its receptor densities differ between leucocyte subsets and exposure to dopamine affects the subsets differently. Overall, dopamine appears to increase natural-killer activity, and inhibit T-lymphocyte and neutrophil function. Thus, the leukocyte subset count associations with facets of IP do not fit with surrogacy of cell function for dopaminergic status, even were count assumed to represent function.

### Crucial future directions

An association of non-*H. pylori* gastric *Helicobacters* with IP [64] would have profound implications as to whether classical autoimmunity or pattern-recognition cross-reactivity is involved in the pathogenesis. Classical autoimmunity may be confined to particular strains within a bacterial species, whilst pattern recognition tends to be shared at genus level.

Whereas spouses of IP-patients have been used as controls, we find they are an important source of pathogenic clues [27,44]. The prevalence of hydrogen-breath-test positivity in spouses was similar to that in IP-patients. Although the number tested was small, it is beyond the balance of probability that the prevalence in spouses is less than 38%. Replication would bring understanding the spouses’ predisposition to the fore in unravelling environmental causality.

More detailed observational work on slow transit, faecal impaction, gut microbiota, and understanding the immune response of the gut (which contains “70% of the immune system” [65]) could identify new targets and strategies to influence disease progression. Cultural approaches to the gut microbiota are being superseded, as research tools, by metagenomic molecular microbiology. This has already defined patterns of stool microbiota (enterotypes) in the population and indicated changes in the spectrum in inflammatory bowel disease [66-68]. These changes include under representation of Gram positive anaerobes (a major component of faecal microbiota in health), which inhibit nuclear factor κB-related proinflammatory cytokine expression [66] and can modulate effects of regulatory T-cells on tolerance and autoimmunity [69]. The loss may be counterbalanced by increased representation of Gram negative bacteria expressing pro-inflammatory molecules, like lipopolysaccharide. Resort to aspiration, brushings or biopsy may be necessary to characterise proximal and distal overgrowth. Further work could, by defining the enterotype in IP with and without *Helicobacter*, home in on candidate drivers of systemic inflammatory activation leading to neuronal damage.

## Conclusion

With respect to Hill’s [56] classic attributes of a causal relationship, we have a coherent, hierarchical and biological-plausible scheme for the pathogenesis of idiopathic parkinsonism, with biological gradients and a proposed time sequence of events. Interpretation may be wrong in parts, due to real gaps in knowledge or lack of awareness of alternative explanations. However, the overall thrust gives new insights into potentially-remediable pathogenic influences. Hill’s attributes of a causal relationship also include strength of key associations expressed as relative risk or odds ratios, experimental evidence and analogies. These are well represented in the work-up. His attribute of consistency through repetition by different groups, in a range of locations selected to test robustness, needs addressing. That of specificity ‘in cause’ should be treated cautiously, since the scheme evokes more than one driving factor. Moreover, specificity may be masked, in a disease with a long prodrome, by classifying as ‘unaffected’ or true negatives those progressing towards the diagnostic threshold [17,27,44] and/or in whom the causal factor will first manifest as another condition (eg. prodromal peptic ulceration in IP).

## Competing interests

The authors declare no competing financial interests.

## Authors’ contributions

RJD, SMD, AC, CW, IB and DT designed research; RJD, SMD, CW, MAAI, IB, AJL, OI, CS and JMP performed research; AC, RJD, SMD and CW analyzed data; SMD, RJD, CW and AC wrote paper. All have read and approved the final manuscript.

## Supplementary Material

Additional file 1 **Table S3.** Multivariable models for brady/hypokinesia and rigidity. Click here for file

Additional file 2 **Table S4.** Multivariable models for mean arterial pressure. Click here for file

Additional file 3 **Table S5.** Multivariable models for tremor whilst seated. Click here for file
