# Supplementary material for: Leukocyte-subset counts in idiopathic parkinsonism provide clues to a pathogenic pathway involving small intestinal bacterial overgrowth. A surveillance study
Source: Gut Pathog. 2012 Oct 19;4:12. doi: 10.1186/1757-4749-4-12 (PMC3500215; doi:10.1186/1757-4749-4-12)
Supplement: Additional file 1 — Table S3. Multivariable models for brady/hypokinesia and rigidity. [file 1757-4749-4-12-S1.doc]

**Table 3. Multivariable models for brady/hypokinesia and rigidity.**

1. in core group (those receiving no anti-parkinsonian medication or background medication other than levodopa: n=38, 265 outcome observations).

| Outcome measure | Natural-killer count | | T-helper count | | | Covariates^a^ |  | |
| --- | --- | --- | --- | --- | --- | --- | --- | --- |
|  | Estimated size effect  per 100 cells.μl^-1^ increment  Mean (95% CI) | *p*-value | | Estimated size effect  per 100 cells.μl^-1^ increment  Mean (95% CI) | *p*-value |  | Estimated size effect/unit increment in outcome  Mean (95% CI) | *p*-value |
| Mean stride-length (mm)^b^ | -49 (-85, -14) | 0.007 | | - | - | Background medication (no=0; yes=1)  Age (year)  Height (mm) | -152 (-244, -59)  -7 (-13, -2)  9 (4, 13) | 0.001  0.007  0.001 |
| Free-walking-speed (mm.s^-1^)^c^ | -54 (-104, -3) | 0.04 | | - | - | Background medication | -283 (-409,-157) | 0.001 |
| Flexor-rigidity (Nm.10^-3^)^d^ | 89 (2, 177) | 0.04 | | -36 (-63, -10) | 0.007 | Time since diagnosis (year) | 39 (22, 56) | 0.001 |
| Extensor-rigidity (Nm.10^-3^) | - | - | | -15 (-27, -3) | 0.02 | Time since diagnosis  Body weight (kg) | 24 (14, 34)  4 (0, 8) | 0.001  0.05 |

^a^Whilst demographic covariates reaching significance at 0 .05 level are tabulated, other covariates (for^b,c,d^) were included in models because of size of effect/pre-determined relevance:-

^b^Time since diagnosis: -7 (-15, 1) mm/year, *p*=0.09. (Effect of anti-nuclear antibody seropositivity large (decrease of 118 (361, 2) mm, *p*=0.05), but, since only 3 positive, ANA status excluded from model.)

^c^Age and height: 6 (-14, 1) & 6 (-1, 12) mm/s, respectively, *p*=0.1 in each case.

^d^Background medication: 167 (-47, 381) Nm x 10^-3^, *p*=0.1).

1. **in untreated group (those not receiving anti-parkinsonian** medication: n=17, 166 outcome observations).

| Outcome measure | Natural-killer count | | T-helper count | | Covariates |  |  |
| --- | --- | --- | --- | --- | --- | --- | --- |
|  | Estimated size effect  per 100 cell.μl^-1^ increment Mean (95% CI) | *p*-value | Estimated size effect  per 100 cells.μl^-1^ increment  Mean (95% CI) | *p*-value |  | Estimated size effect/unit increment in outcome  Mean (95% CI) | *p*-value |
| Mean stride-length | -59 (-103, -15) | 0.009 | - | - | Age | -12 (-18, -5) | 0.001 |
| Free-walking-speed | -73 (-130, -15) | 0.01 | - | - | Age | -12 (-21, -3) | 0.005 |
| Flexor-rigidity | 95 (36, 154) | 0.001 | -42 (-61, -22) | 0.001 | Time since diagnosis | 49 (27, 70) | 0.001 |
| Extensor-rigidity | - | - | -13 (-24, -2) | 0.02 | Time since diagnosis  Body weight | 15 (4, 27)  8 (3, 12) | 0.01  0.001 |

1. **in entire group (including those receiving levodopa: n=51, 362 outcome observations).**

| Outcome measure | Natural-killer count | | T-Helper count | | Covariates |  |  |
| --- | --- | --- | --- | --- | --- | --- | --- |
|  | Estimated size effect  per 100 cell.μl^-1^ increment  Mean (95% CI) | *p*-value | Estimated size effect  per 100 cells.μl^-1^ increment  Mean (95% CI) | *p*-value |  | Estimated size effect/unit increment in outcome  Mean (95% CI) | *p*-value |
| Mean stride-length | -37 (-76, 2)* | 0.06 | - | - | Time since diagnosis  Background medication  Height | -10 (-18, -2)  -135 (-265, -6)  9 (3, 16) | 0.02  0.04  0.006 |
| Free-walking-speed | -49 (-96, -2) | 0.04 | - | - | Background medication | -266 (-398, -133) | 0.001 |
| Flexor-rigidity | - | - | -37 (-64, -10) | 0.008 | Time since diagnosis | 20 (5, 35) | 0.008 |
| Extensor-rigidity | - | - | -14 (-26, -2) | 0.02 | Time since diagnosis  Body weight | 16 (8, 24)  5 (2, 9) | 0.001  0.003 |

* since size of effect mirrored that in a) & b), value given although probability >0.05.
