# Supplementary material for: Leukocyte-subset counts in idiopathic parkinsonism provide clues to a pathogenic pathway involving small intestinal bacterial overgrowth. A surveillance study
Source: Gut Pathog. 2012 Oct 19;4:12. doi: 10.1186/1757-4749-4-12 (PMC3500215; doi:10.1186/1757-4749-4-12)
Supplement: Additional file 2 — Table S4. Multivariable models for mean arterial pressure. [file 1757-4749-4-12-S2.doc]

**Table 4. Multivariable models for mean arterial pressure.**

1. **in core group**

| Outcome measure | Natural-killer count | | Covariates |  | |
| --- | --- | --- | --- | --- | --- |
|  | Estimated size effect  per 100 cells.μl^-1^ increment  Mean (95% CI) | *p-*value |  | Estimated size effect  per unit increment in outcome  Mean (95% CI) | *p*-value |
| Supine mean arterial pressure (mm Hg) | 4.2 (1.2, 7.2) | 0.007 | Age (year)  Time since diagnosis (year)  Body weight (kg) | 0.4 (0, 0.9)  -1.1 (-1.8, -0.3)  0.4 (0.1, 0.7) | 0.05  0.005  0.02 |

1. **in untreated group**

| Outcome measure | Natural-killer count | | Covariates |  | |
| --- | --- | --- | --- | --- | --- |
|  | Estimated size effect  per 100 cells.μl^-1^ increment  Mean (95% CI) | *p*-value |  | Estimated size effect  per unit increment in outcome  Mean (95% CI) | *p*-value |
| Supine mean arterial pressure | 2.8 (-1.6, 7.3) | 0.2* | Age | 0.7 (0.1, 1.4) | 0.03 |

*since size of effect, in b), mirrored that in c), values given although probability >.05.

1. in entire group

| Outcome measure | Natural-killer count | | Covariates |  | |
| --- | --- | --- | --- | --- | --- |
|  | Estimated size effect  per 100 cells.μl^-1^ increment  Mean (95% CI) | *p*-value |  | Estimated size effect  per unit increment in outcome  Mean (95% CI) | *p*-value |
| Supine mean arterial pressure | 3.0 (0.7, 5.4) | 0.01 | Age | 0.5 (0.2, 0.9) | 0.006 |
