# Supplementary material for: Leukocyte-subset counts in idiopathic parkinsonism provide clues to a pathogenic pathway involving small intestinal bacterial overgrowth. A surveillance study
Source: Gut Pathog. 2012 Oct 19;4:12. doi: 10.1186/1757-4749-4-12 (PMC3500215; doi:10.1186/1757-4749-4-12)
Supplement: Additional file 3 — Table S5. Multivariable models for tremor whilst seated. [file 1757-4749-4-12-S3.doc]

**Table 5. Multivariable models for tremor whilst seated.**

1. **in core group**

| Outcome measure | Neutrophil count | | Covariates | |  |  | |
| --- | --- | --- | --- | --- | --- | --- | --- |
|  | Estimated size effect  per 10^9^ cells.l^-1^ increment  Mean (95% CI) | *p-*value |  | Estimated size effect  per unit increment in outcome  Mean (95% CI) | | | *p-*value |
| Mean tremor  (100 none, 0 worst) | 6.2 (1.2, 11.1) | 0.01 | Time since diagnosis (year) | -1.2 (-2.2, -0.02) | | | 0.02 |
| at rest | 5.6 (1.0, 1.3) | 0.02 | Time since diagnosis | -1.7 (-3.0, -0.4) | | | 0.009 |
| under stress | 6.1 (0.3, 11.9) | 0.04 | - | - | | | - |

1. **in untreated group**

| Outcome measure | Neutrophil count | | Covariates | |  |  | |
| --- | --- | --- | --- | --- | --- | --- | --- |
|  | Estimated size effect  per 10^9^ cells.l^-1^ increment  Mean (95% CI) | *p-*value |  | Estimated size effect  per unit increment in outcome  Mean (95% CI) | | | *p-*value |
| Mean tremor | 4.6 (-1.3, 1.5)* | 0.1 | Time since diagnosis | -1.5 (-2.8, -0.2) | | | 0.03 |
| at rest | 4.2 (-1.1, 9.5)* | 0.1 | Time since diagnosis | -2.1 (-3.8, -0.4) | | | 0.02 |
| under stress | 4.7 (-2.0, 11.5)* | 0.1 | - | - | | | - |

*since size of effect, in b) & c), mirrored that in a), values given although probability >.05.

1. in entire group

| Outcome measure | Neutrophil count | | Covariates | |  |  | |
| --- | --- | --- | --- | --- | --- | --- | --- |
|  | Estimated size effect  per 10^9^ cells.l^-1^ increment  Mean (95% CI) | *p-*value |  | Estimated size effect  per unit increment in outcome  Mean (95% CI) | | | *p-*value |
| Mean tremor | 4.5 (0.2, 8.8) | 0.04 | - | - | | | - |
| at rest | 4.2 (0.2, 8.1) | 0.04 | Time since diagnosis | -1.2 (-2.3, -0.1) | | | 0.03 |
| under stress | 4.3 (-0.6, 9.2)* | 0.09 | - | - | | | - |
